# Supplementary material for: Quantum walks and Dirac cellular automata on a programmable trapped-ion quantum computer
Source: Nat Commun. 2020 Jul 24;11:3720. doi: 10.1038/s41467-020-17519-4 (PMC7381638; doi:10.1038/s41467-020-17519-4)
Supplement: Supplementary file 1 — Supplementary Information [file 41467_2020_17519_MOESM1_ESM.pdf]

Reviewers' Comments:

Reviewer #1:
Remarks to the Author:
The main result claimed in the paper under review is an experimental realization of a DQW on five qubits within a seven-qubit programmable trapped-ion quantum computer. Further, the DQW realization is tuned to carry out a simulation of a Dirac equation-like dynamics.

The paper commences with a review of quantum walks and the Dirac equation. This material is by now standard. This is followed with a description of the experimental implementation where a mapping of the particle position variable to various Hilbert space states of the 7-qubit simulator is detailed. The simulator itself comprises seven Yb ions in a Paul trap, of which only five are used to encode qubits. The required gate sequence to simulate a DQW is also detailed. After 3000 steps the probability distribution is sampled. Experimental results consistent with a variety of effective masses are described. Typical bimodal distributions are observed. The paper ends with the conclusion that DQW can simulate DCA, despite population mismatches.

It is important to state at this point that, as a theorist, I am *not at all* qualified to assess the experimental aspects of this paper. Therefore I restrict my comments in the following to the theoretical aspects alone. I openly admit that this likely means my report is dramatically biased. I do hope an experimental referee can meaningfully complement my assessment.

Concerning the theoretical implications of this paper: I looked through the paper to find novel theoretical constructions/results. The decisive question here is what value a 5-qubit simulator brings, given that classical computers can routinely simulate the full dissipative non-Markovian dynamics of quantum systems with 2^5 = 32 levels. The most natural response here would be that it is vital to understand the actual noise occurring in experiments, and compare them with theoretical predictions. There is some discussion here in the paper of this point, but the results are restricted somewhat to a simplistic side-by-side comparison of ideal(?) noiseless numerics and the experimentally measured probability distributions. This really isn't enough to identify and assess the validity of a noise model, Markovian or otherwise. With a 32 x 32 density matrix it ought to be possible to do some kind of tomography and go some way to identifying a realistic noise model. The conclusion that a 32-level system can simulate a DCA is not theoretically novel. (Indeed, this would be at the level of a graduate quantum exercise.)

For the above-mentioned reasons I cannot recommend publication. If, however, the experimental implementation is novel and interesting I would be very happy to defer to the (presumably positive assessment of) an experimental referee.

Minor comments:

1. In the discussion concerning the relationship with the Dirac equation I would caution against assertions such as "By controlling the parameters θ1 and θ2, the split-step quantum walk turns into the one-dimensional
Dirac equations...". This is not correct: the Dirac equation concerns a particle with a continuous position variable. Perhaps better would be to use a qualifier something like "effectively, within the low momentum subspace", or similar.

2. The experimental results are compared(?) with the continuum solution of the Dirac equation. I would prefer the experimental results be directly compared with the actual unitary evolution of the 32-site DCA, or better still, with the solution of Lindblad equation or similar modelling the noise.

Reviewer #2:

Remarks to the Author:
In their manuscript, Huerta Alderete et al. show the realization of a discrete-time quantum walk (DTQW) in an ion-based quantum processor, in which the position space is mapped into logical states of the processor. With respect to "analog" implementations of DTQW, in which the quantum walk emerges as an evolution of particles in a physical space (e.g. photons in a photonic circuit), a "quantum circuit" implementation of DTQW has the advantages of making it easier to change the experimental parameters and, most importantly, to include quantum walk processes in more complex computational procedures.

The "quantum circuit" approach in realizing quantum walks is not new (see Ref.s 36 and 37), but the manuscript reports its first realization on an ion-based quantum hardware. This achievement is certainly interesting for the broad audience of Nature Communications, as ion-based quantum computers are a powerful tool in quantum technologies. However, the manuscript lacks of clarity on a few points, which I believe should be addressed by the authors.

My main concern regards possible intrinsic limitations of the protocol. In Table 1, the authors show the logical encoding of the position space into internal states of the ions. There, the state|1000> is not part of the mapping, but – I believe – is part of the evolution of the quantum processor. As a result, the DTQW occurs in a subspace of the processor's Hilbert space. Is this limitation causing a systematic error in the quantum walk? Is this a systematic error that can somehow be corrected? Does this possible error cause a stronger deviation between theory and experiment for a number of steps larger than 5 (the number of steps shown in the manuscript)? Additionally, I wonder if there are limitations on the circuital approach proposed by the authors. Can for instance the evolution begin from any location in the position space, or must the evolution begin from the center (position 0)?

Moreover, I have a few minor remarks:

a. The methods mention Fig.s B and C (surprisingly, there is no Fig. A), but I cannot see them in the manuscript
b. For better clarity, I would add a "c" subscript on all the kets referring to the coin state.

Reviewer #3:
Remarks to the Author:
In their manuscript "Quantum walks and Dirac cellular automata on a programmable trapped-ion quantum computer", the authors describe the implementation of circuit based quantum walks. As the authors correctly describe in the introduction, quantum walks are important for modeling quantum systems, simulations of the Dirac equation and for developing a wide range of quantum algorithms. Trapped ions are a leading platform in the development of quantum devices owing to the superb quality of operations they offer. This makes the topic of the work of immediate relevance for emerging quantum technology. Given the scope and relevance of developing these technologies, the work is suitable for a general readership journal such as NC.

Both quantum simulations of the Dirac equation and quantum walks have been implemented in trapped ions before by encoding the "position" and "momentum" of the walker/relativistic particle in the amplitude and phase of the oscillations of the ion in its trap, e.g. [refs 33-35]. What is new in the present work is that the quantum walk is done in a quantum circuit, i.e. the position of the walker is encoded in the qubit states of the ions. This is a significant step forward, as exploring the algorithm applications based on QWs would require a circuit rather than an analogue simulation based on continuous variables of ion motion.

In my opinion, this is excellent work. The level of control and accuracy of the quantum operations is truly amazing and the manuscript is very well written. I warmly recommend this manuscript for

publication in NC. I just have a few minor comments:
- It would be good in my opinion to state very clearly at the location where the Dirac equation is introduced that it is in fact the Dirac equation in 1 dimension (i.e. no spin degree of freedom). The general readership may otherwise be confused with the 2x2 matrices used instead of the usual 4x4.
- It is not so clear to me what the outlook of the simulated Dirac equation dynamics is. While I see that a circuit based approach promises is more scalable than the work done by the Innsbruck group on the Zitterbewegung and the Klein paradox, it seems to me that the Dirac equation is an inherently one-particle theory. Or could the formalism developed by the authors be used to e.g. also study quantum field theories? A bit more outlook on this aspect would improve the paper in my opinion.

Reviewer #1 (Remarks to the Author):

The main result claimed in the paper under review is an experimental realization of a DQW on five qubits within a seven-qubit programmable trapped-ion quantum computer. Further, the DQW realization is tuned to carry out a simulation of a Dirac equation-like dynamics.

The paper commences with a review of quantum walks and the Dirac equation. This material is by now standard. This is followed with a description of the experimental implementation where a mapping of the particle position variable to various Hilbert space states of the 7-qubit simulator is detailed. The simulator itself comprises seven Yb ions in a Paul trap, of which only five are used to encode qubits. The required gate sequence to simulate a DQW is also detailed. After 3000 steps the probability distribution is sampled. Experimental results consistent with a variety of effective masses are described. Typical bimodal distributions are observed. The paper ends with the conclusion that DQW can simulate DCA, despite population mismatches.

It is important to state at this point that, as a theorist, I am *not at all* qualified to assess the experimental aspects of this paper. Therefore I restrict my comments in the following to the theoretical aspects alone. I openly admit that this likely means my report is dramatically biased. I do hope an experimental referee can meaningfully complement my assessment.

Concerning the theoretical implications of this paper: I looked through the paper to find novel theoretical constructions/results.

We want to thank the reviewer for the careful reading of our manuscript. The relationship between quantum walks/quantum cellular automaton in one-dimensional space and the Dirac equation is a well established theoretical result. The value of our contribution relies on the circuit implementation of QWs and DCA on a trapped-ion processor via an efficient mapping of qubit states to walker position states, which allows us to accomplish a high simulation performance despite going to deep quantum circuits.

The decisive question here is what value a 5-qubit simulator brings, given that classical computers can routinely simulate the full dissipative non-Markovian dynamics of quantum systems with 2^5 = 32 levels.

It is true that classical computers can routinely simulate the dynamics of a quantum system with 5 or more qubits. Classical simulation of a large number of steps of QW has also been reported. QWs are a quantum algorithm able to simulate many quantum systems/models and various quantum computation tasks effectively on a quantum machine. In this work, we have tried to map QW onto a quantum circuit and then used it to simulate Dirac cellular automata on a quantum processor. As pointed out by the other reviewers, this is the first scalable realization of a circuit-based quantum walk and a start to show that quantum circuits, by controlling the

evolution parameters, can be effectively used to simulate any quantum model that has been engineered using QWs.

The most natural response here would be that it is vital to understand the actual noise occurring in experiments, and compare them with theoretical predictions. There is some discussion here in the paper of this point, but the results are restricted somewhat to a simplistic side-by-side comparison of ideal(?) noiseless numerics and the experimentally measured probability distributions. This really isn't enough to identify and assess the validity of a noise model, Markovian or otherwise. With a 32 x 32 density matrix it ought to be possible to do some kind of tomography and go some way to identifying a realistic noise model. The conclusion that a 32-level system can simulate a DCA is not theoretically novel. (Indeed, this would be at the level of a graduate quantum exercise.)

Our system is a fully connected trapped ion quantum computer where the main source of error is related to intensity fluctuations on the Raman beams and cooling imperfections causing an average state detention fidelity for single- and two-qubit gates of 99.5% and 98-99%, respectively. We find circuit behavior consistent with these errors on this noisy prototype device, which means that larger-scale simulations will come into reach as the hardware improves. Quantum simulators are only now starting to challenge classical simulations and we expect circuit based quantum computers for follow suit.

For the above-mentioned reasons I cannot recommend publication. If, however, the experimental implementation is novel and interesting I would be very happy to defer to the (presumably positive assessment of) an experimental referee.

Minor comments:

1. In the discussion concerning the relationship with the Dirac equation I would caution against assertions such as "By controlling the parameters θ_1 and θ_2, the split-step quantum walk turns into the one-dimensional Dirac equations...". This is not correct: the Dirac equation concerns a particle with a continuous position variable. Perhaps better would be to use a qualifier something like "effectively, within the low momentum subspace", or similar.

We rephrase the sentence, now it reads:

> “The tunability of parameters θ_{1} and θ_{2} on the split-step QW permits the study of one-dimensional Dirac equations effectively, within the low momentum subspace, for spin-$1/2$ particles \cite{Mallick2016p25779, Kumar2016p012116}.”

2. The experimental results are compared(?) with the continuum solution of the Dirac equation. I would prefer the experimental results be directly compared with the actual unitary evolution of the 32-site DCA, or better still, with the solution of Lindblad equation or similar modelling the noise.

The results are indeed compared to the actual unitary evolution of the 32-site DCA in figure 2. We simulated the unitary evolution given by $\hat{W}=\hat{S}\hat{C}_{\theta}$ (32-site system) and traced out the coin information to show the position of the walker after each time-step. For clarity, the appendix gives the difference between the experimental results and this evolution on a finer scale. While we don't present a Lindblad treatment, the increasing mismatch is consistent with the accumulation of gate errors as the simulation moves to deeper quantum circuits. We now state this explicitly in the text:

> "In order to illustrate how our experiment performs, we plot the absolute value of the difference between measured and simulated position distributions, Fig. \ref{fig:error}, they match the theoretical expectation closely. These distributions are obtained after tracing out the coin information of the unitary evolution $\hat{W}=\hat{S}\hat{C}_{\theta}$ for each time-step. In both instances, DQW and DCA, the number of gates and hence the error incurred grows with the number of steps."

> As a final comparison, in Methods C, we show the numerical simulation of the explicit time-dependent solution of the one-dimensional Dirac equation and the unitary evolution of DCA to show that this method reproduces the result given in Phys. Rev. A 73 054302 (2006).

Reviewer #2 (Remarks to the Author):

In their manuscript, Huerta Alderete et al. show the realization of a discrete-time quantum walk (DTQW) in an ion-based quantum processor, in which the position space is mapped into logical states of the processor. With respect to "analog" implementations of DTQW, in which the quantum walk emerges as an evolution of particles in a physical space (e.g. photons in a photonic circuit), a "quantum circuit" implementation of DTQW has the advantages of making it easier to change the experimental parameters and, most importantly, to include quantum walk processes in more complex computational procedures.

The "quantum circuit" approach in realizing quantum walks is not new (see Ref.s 36 and 37), but the manuscript reports its first realization on an ion-based quantum hardware. This achievement is certainly interesting for the broad audience of Nature Communications, as ion-based quantum computers are a powerful tool in quantum technologies. However, the manuscript lacks of clarity on a few points, which I believe should be addressed by the authors.

My main concern regards possible intrinsic limitations of the protocol. In Table 1, the authors show the logical encoding of the position space into internal states of the ions. There, the state|1000> is not part of the mapping, but – I believe – is part of the evolution of the quantum processor. As a result, the DTQW occurs in a subspace of the processor's Hilbert space. Is this

limitation causing a systematic error in the quantum walk? Is this a systematic error that can somehow be corrected?. Does this possible error cause a stronger deviation between theory and experiment for a number of steps larger than 5 (the number of steps shown in the manuscript)?

We want to thank the reviewer for the careful reading of our manuscript and pointing out the relevance of our work for the audience of Nature communications. We are considering a symmetric quantum walk about |x=0> within a space of 2^n -1 positions, with n the number of qubits. While this makes very efficient use of the available Hilbert space, there is one remaining state in the logical encoding, |1000>, which we have not included. It could be identified with position 2^(n-1) if considering more steps. It is true that this state can be populated in error during the evolution due to channel-to-channel crosstalk in gate addressing and qubit readout. Our results show that the likelihood of this error occurring is very small. In fact, the average experimental population registered in this state is <2% for the deepest circuits and hence does not affect the results significantly.
Now, we have included on Methods B:
"(...) The same mechanism can populate the state $\vert 1000 \rangle$ of the logical encoding not included in our mapping. In fact, the average experimental population registered in this state is $<2\%$ for the deepest circuits and hence does not affect the results significantly. "

Additionally, I wonder if there are limitations on the circuital approach proposed by the authors. Can for instance the evolution begin from any location in the position space, or must the evolution begin from the center (position 0)?

The state-mapping we have chosen is not unique but makes optimal use of the Hilbert space available. By fixing the initial state, the quantum circuit complexity as well as the error rate can be reduced. We have chosen to start the system in the center in order to allow the widest possible distribution of a given qubit number.
We now state this in the manuscript:

> "We note that the particle can be started from any point in the position space, however fixing the initial state reduces the gate counting in the circuit, and hence the overall error."

Moreover, I have a few minor remarks:

a. The methods mention Fig.s B and C (surprisingly, there is no Fig. A), but I cannot see them in the manuscript

There must have been an error in the transmission of the manuscript, we apologize the figure didn't appear. The methods section has four subsections labelled A-D and 3 figures labelled numerically. The first figure shows the experimental errors, the second a comparison of the DCA with an analytical solution, and the third gives the quantum gate compilation used to express the

high-level circuits.

b. For better clarity, I would add a “c” subscript on all the kets referring to the coin state.

We followed the reviewer’s suggestion and added a ‘c’ subscript to all kets referring to the coin state.

Reviewer #3 (Remarks to the Author):

In their manuscript “Quantum walks and Dirac cellular automata on a programmable trapped-ion quantum computer”, the authors describe the implementation of circuit based quantum walks. As the authors correctly describe in the introduction, quantum walks are important for modeling quantum systems, simulations of the Dirac equation and for developing a wide range of quantum algorithms. Trapped ions are a leading platform in the development of quantum devices owing to the superb quality of operations they offer. This makes the topic of the work of immediate relevance for emerging quantum technology. Given the scope and relevance of developing these technologies, the work is suitable for a general readership journal such as NC.

Both quantum simulations of the Dirac equation and quantum walks have been implemented in trapped ions before by encoding the “position” and “momentum” of the walker/relativistic particle in the amplitude and phase of the oscillations of the ion in its trap, e.g. [refs 33-35]. What is new in the present work is that the quantum walk is done in a quantum circuit, i.e. the position of the walker is encoded in the qubit states of the ions. This is a significant step forward, as exploring the algorithm applications based on QWs would require a circuit rather than an analogue simulation based on continuous variables of ion motion.

In my opinion, this is excellent work. The level of control and accuracy of the quantum operations is truly amazing and the manuscript is very well written. I warmly recommend this manuscript for publication in NC.

We want to thank the reviewer for the positive assessment of our manuscript.

I just have a few minor comments:
- It would be good in my opinion to state very clearly at the location where the Dirac equation is introduced that it is in fact the Dirac equation in 1 dimension (i.e. no spin degree of freedom). The general readership may otherwise be confused with the 2x2 matrices used instead of the usual 4x4.

This was also pointed out by reviewer 1. We rephrased the sentence, now it reads:

> “The tunability of parameters θ_{1} and θ_{2} on the split-step QW permits

the study of one-dimensional Dirac equations effectively, within the low momentum subspace, for spin-\$1/2\$ particles \cite{Mallick2016p25779, Kumar2016p012116}. It is important to stress out that the description of the Dirac equation used here corresponds to the \$2\times2\$ representation, i. e. no spin degree of freedom."

- It is not so clear to me what the outlook of the simulated Dirac equation dynamics is. While I see that a circuit based approach promises is more scalable than the work done by the Innsbruck group on the Zitterbewegung and the Klein paradox, it seems to me that the Dirac equation is an inherently one-particle theory. Or could the formalism developed by the authors be used to e.g. also study quantum field theories? A bit more outlook on this aspect would improve the paper in my opinion.

We agree with the referee that there are broader applications to consider. We have added the following to the manuscript.

"The correspondence between DQWs and the dynamics of Dirac particles suggests that the QWs formalism is as a viable approach to reproduce a variety of phenomena underpinned by Dirac particle dynamics in both the high- and low-energy regime \cite{Mallick2017p85, Flurin2017p031023, Basio2015p244}. Quantum simulations of free quantum field theory \cite{Basio2015p244}, Yang-Mills gauge-field on fermionic matter \cite{Arnault2016p012335}, as well as the effect of mass and space-time curvature on entanglement between accelerated particles \cite{Arrighi2018p84, Mallick2019p0150012, Singh2019p055008} have been reported and probing quantum field theory from the algorithmic perspective in an active field of research. However, the circuit complexity for position-dependent coin operations needed for simulating these effects will increase with the complexity of the evolution, which means further improvements in quantum hardware will be necessary for their realization."
